# Integrated Diagnosis of Hepatitis B, C, and D Viruses and HIV in Populations Evaluated for Sexually Transmitted Infections: A Narrative Review and Operational Framework

**DOI:** 10.3390/v18080861

**Published:** 2026-08-06

**Authors:** Joaquín Cabezas, Ezequiel Ridruejo, José Antonio Velarde-Ruiz Velasco, Graciela Castro-Narro, Lorena Cayón-González, Carolina Jiménez, Hugo Cheinquer, Fernando Contreras, Nelia Hernández, Christie Perelló, José Luis Calleja, Javier Crespo

**Affiliations:** 1 Gastroenterology and Hepatology Department, Hospital Universitario Marqués de Valdecilla, 39008 Santander, Spain; 2 Clinical and Translational Research in Digestive Diseases, Valdecilla Research Institute (IDIVAL), 39008 Santander, Spain; 3 Hepatology Section, Department of Medicine, Centro de Educación Médica e Investigaciones Clínicas Norberto Quirno (CEMIC), Ciudad Autónoma de Buenos Aires C1425ASG, Argentina; eridruejo@gmail.com; 4 Servicio de Gastroenterología, Hospital Civil de Guadalajara Fray Antonio Alcalde, Guadalajara 44280, Mexico; 5 Unidad de Hepatología y Trasplante, Hospital Médica Sur, Ciudad de México 14050, Mexico; 6 Departamento de Gastroenterología, Hepatología y Trasplante, Instituto Nacional de Ciencias Médicas y Nutrición Salvador Zubirán, Ciudad de México 14080, Mexico; 7 Departamento de Medicina Interna, Facultade de Medicina, Universidade Federal do Rio Grande do Sul, Porto Alegre 90040-060, Brazil; hcheinquer@gmail.com; 8 Hepatology Department, Hospital de Clínicas de Porto Alegre, Porto Alegre 90035-903, Brazil; 9 Centro de Gastroenterología Avanzada de Santo Domingo, Santo Domingo 10148, Dominican Republic; 10 Gastroenterology Academic Unit, Hospital de Clínicas, Faculty of Medicine, Universidad de la República (UdelaR), Montevideo 11200, Uruguay; 11 Unidad de Hepatología, Centro de Estudios Digestivos, Hospital Metropolitano Santiago (HOMS), Santiago de los Caballeros 51000, Dominican Republic; 12 Department of Gastroenterology and Hepatology, Hospital Universitario Puerta de Hierro, Universidad Autónoma de Madrid, 28222 Madrid, Spain; 13 Valdecilla Research Institute (IDIVAL), Universidad de Cantabria, 39008 Santander, Spain; 14 MedicineAI Association, 28027 Madrid, Spain

**Keywords:** hepatitis B, hepatitis C, hepatitis D, HIV, sexually transmitted infections, reflex testing, microelimination, PrEP, chemsex, bulevirtide

## Abstract

Hepatitis B virus (HBV), hepatitis C virus (HCV), and hepatitis D virus (HDV), together with HIV and bacterial sexually transmitted infections (STIs), account for a rising toll of more than one million deaths annually and overlap within shared behavioral and social networks, yet are still diagnosed through separate, pathogen-specific pathways. This narrative review synthesizes contemporary evidence on the convergence between viral hepatitis, HIV, and high-risk STI groups—men who have sex with men, pre-exposure prophylaxis (PrEP) users, people practicing chemsex, people who inject drugs, incarcerated populations, and migrants from endemic regions—and characterizes the diagnostic technologies and linkage-to-care models available to address it. Despite effective antivirals, the diagnostic cascade remains the principal bottleneck: most people with chronic HCV are undiagnosed, and HDV—with a pooled anti-HDV seroprevalence of approximately 4.5% among HBsAg-positive individuals—is its clearest expression, as most carriers are never tested. Reflex algorithms, multiplex panels, point-of-care assays, dried blood spots, and electronic health record alerts are validated but unevenly implemented. We argue that, alongside persistent resource, political, and equity-related constraints, a substantial share of the remaining barriers is operational rather than purely technological, and propose a practical, STI-clinic-centered framework integrating reflex testing, population-specific periodicity, and explicit linkage pathways toward the WHO 2030 elimination targets.

## 1. Introduction

Despite highly effective antiviral regimens, viral hepatitis and HIV remain leading global public health challenges. Chronic HBV (hepatitis B virus) and HCV (Hepatitis C virus) together accounted for more than 1.1 million deaths per year in earlier estimates, and the burden has continued to rise: the WHO Global Hepatitis Report 2026 attributes 1.34 million deaths in 2024 to hepatitis B and C, with approximately 287 million people living with chronic HBV or HCV. Polaris Observatory projections indicate that most countries remain off-track for the World Health Organization (WHO) 2030 elimination targets—90% of infected individuals diagnosed, 80% of those eligible treated, and a 65% reduction in liver-related mortality [1,2,3]. HIV and bacterial STIs continue to expand in several high-income regions, disproportionately affecting populations exposed to overlapping sexual, behavioral, and structural risk factors [4,5]. Across all four pathogens, persistent under-diagnosis remains the principal barrier to elimination, with global cascade analyses estimating that of approximately 57 million people with chronic HCV in 2020, only ~12.9 million had been diagnosed and ~641,000 initiated treatment that year, well below WHO 2030 targets [6,7].

Historically, viral hepatitis, HIV, and classical STIs have been managed through separate diagnostic, clinical, and organizational pathways. This compartmentalized model is increasingly outdated. Substantial evidence documents epidemiological convergence between HIV, STIs, and viral hepatitis—particularly among men who have sex with men (MSM), individuals engaged in chemsex, HIV pre-exposure prophylaxis (PrEP) users, sex workers, incarcerated populations, and migrants from endemic regions [8,9]. In these groups, infections cluster within interconnected networks characterized by repeated exposure, reinfection after cure, high concomitant STI burden, and shared structural vulnerabilities, well captured by the syndemic framework [10,11].

The recognition of sexually transmitted HCV among MSM marked a defining epidemiological shift. Early European cohorts identified outbreaks of acute HCV in HIV-positive MSM in the absence of injection drug use, and subsequent phylogenetic analyses revealed large international transmission clusters across Europe and Australia [12,13]. The expansion of PrEP further confirmed that HCV sexual transmission is not confined to HIV-positive populations: cohorts from the Netherlands, France, Australia, and Spain reported substantial HCV incidence among HIV-negative PrEP users alongside high rates of bacterial STIs and recurrent exposure [14,15]. Reinfection after virological cure, with rates approaching or exceeding primary incidence in some cohorts of HIV-positive MSM, decisively challenges the single-event model of HCV diagnosis [16,17].

The overlap is not limited to HCV. Globally, an estimated 257.5 million people were living with chronic HBV (3.2% prevalence) in 2022 [2], a figure updated to approximately 240 million in 2024 by the WHO Global Hepatitis Report 2026 [3]; the highest burden remains in West Africa and the Western Pacific, and HBV remains highly prevalent among MSM, migrants from endemic regions, and incarcerated populations [18,19,20]. HDV, the most severe form of chronic viral hepatitis, remains profoundly under-diagnosed despite the approval of bulevirtide. Pooled global anti-HDV seroprevalence among HBsAg-positive individuals has been estimated at approximately 4.5%, with updated modeling suggesting a worldwide HDV-infected population in the order of tens of millions, although recent adjusted analyses indicate that historical estimates may have been over- or under-estimated in different regions [21,22,23].

The principal contemporary barriers to elimination lie in implementation rather than in diagnostic capability. Reflex HCV-RNA and reflex anti-HDV testing, multiplex molecular assays, point-of-care platforms, dried blood spot (DBS) testing, opt-out screening, and electronic health record (EHR) algorithms have been validated in real-world settings [24,25,26]. Linkage-to-care can likewise be reinforced through EHR alerts, navigation, telemedicine, and decentralized pathways [27,28]. Yet implementation remains heterogeneous, even in high-income contexts [29,30]. The WHO Global Health Sector Strategies 2022–2030, together with EASL, EACS, AASLD–IDSA, ECDC, and CDC frameworks, explicitly endorse combined HBV/HCV/HIV testing in vulnerable populations [31,32,33,34].

This narrative review synthesizes contemporary evidence on combined diagnosis of HBV, HCV, HDV, and HIV in populations evaluated for STIs, with three explicit aims: (i) to map the epidemiological and clinical convergence between viral hepatitis and STI-associated populations; (ii) to characterize the diagnostic technologies, linkage-to-care models, microelimination strategies, and implementation barriers documented in the literature; and (iii) to propose a practical, STI-clinic-centered operational framework—integrating reflex algorithms, periodicity by population, and explicit linkage pathways—capable of translating elimination strategy into routine clinical workflow. Key concepts and definitions are summarized in Box 1.

Box 1 **Key concepts and operational definitions.**

- Integrated diagnostic ecosystem. Reorganization of viral hepatitis and HIV testing around shared epidemiological risk and the patient’s healthcare trajectory rather than around isolated pathogens, combining serological screening, reflex molecular confirmation, vaccination, prevention, and linkage-to-care within a single clinical workflow.
- Reflex testing. Automated laboratory algorithm that performs a confirmatory or complementary test on the same specimen following a positive screening result (e.g., reflex HCV-RNA after positive anti-HCV; reflex anti-HDV after positive HBsAg; reflex HDV-RNA after positive anti-HDV), eliminating the need for an additional patient encounter.
- Microelimination. Targeted elimination strategies focused on defined subpopulations or healthcare environments (e.g., HIV/PrEP services, prisons, addiction programs) where intensified integrated interventions can achieve measurable outcomes within shorter timeframes than nationwide elimination.
- Syndemic framework. Conceptual model in which HIV, viral hepatitis, STIs, substance use, mental health disorders, stigma, and structural vulnerability interact synergistically rather than additively, amplifying transmission and impairing engagement with care.
- Linkage-to-care. The capacity of healthcare systems to ensure that individuals successfully transition through the full continuum of evaluation and treatment after diagnosis, ideally through same-encounter or same-day pathways supported by EHR alerts, patient navigation, and decentralized care.
- Longitudinal surveillance. Repeated, periodic screening within services with recurrent patient contact (PrEP, STI, addiction, prison), enabling detection of HCV reinfection, HBV reactivation, HDV emergence, and incident HIV/STIs across follow-up.

## 2. Methods

This is a narrative review informed by scoping methodology [35,36], and grounded in implementation-science principles [37]. Consistent with a narrative rather than systematic or scoping review, study identification and selection were guided by author expertise and thematic relevance rather than by a formal protocol; accordingly, no PRISMA-style selection diagram, formal risk-of-bias assessment, or quantitative synthesis was undertaken. We searched PubMed/MEDLINE, Embase, Web of Science Core Collection, and Scopus from 1 January 2010 to 1 March 2026, combining controlled vocabulary (MeSH and Emtree) and free-text terms across three concept blocks: (i) viral hepatitis and HIV (HBV, HCV, HDV, HIV); (ii) STI-associated populations and behaviors (sexually transmitted infections, MSM, PrEP, chemsex, sexual transmission, people who inject drugs, prison, sex workers, migrants); and (iii) combined diagnosis and implementation (combined screening, reflex testing, dried blood spot, point of care, multiplex, opt-out testing, electronic health record, linkage to care, microelimination, implementation). Concept blocks were combined with AND; terms within each block with OR. Because several of the populations central to this review are defined in part by sex, gender, or sexual behavior (e.g., men who have sex with men, transgender persons), findings are reported using the sex and gender categories employed in the source studies; where data were not disaggregated by sex or gender, this is noted.

We additionally hand-searched the reference lists of recent systematic reviews, consensus statements, and guideline documents from the WHO, the European Centre for Disease Prevention and Control (ECDC), the Centers for Disease Control and Prevention (CDC), the European Association for the Study of the Liver (EASL), the European AIDS Clinical Society (EACS), the American Association for the Study of Liver Diseases (AASLD)–Infectious Diseases Society of America (IDSA), and major national scientific societies, including the Spanish Association for the Study of the Liver (AEEH) [15,31,38,39,40]. Hepatology and infectious diseases society guidance was reviewed for HBV, HCV, HDV, and HIV management [32,33,41,42,43,44]. Spanish AEEH guidelines and consensus documents complemented the international literature for the regional implementation context [45,46]. Studies were selected on the basis of relevance to combined diagnosis of HBV, HCV, HDV, and HIV in populations evaluated for STIs. Eligible study types included observational cohorts, cross-sectional studies, outbreak investigations, implementation studies, randomized or quasi-experimental screening interventions, diagnostic-pathway studies, cost-effectiveness analyses, systematic reviews, meta-analyses, consensus documents, and national or international guidelines. Studies focused exclusively on molecular virology without implementation relevance, animal studies, isolated analytical validation studies without real-world applicability, and conference abstracts lacking sufficient methodological detail were not used as primary sources. Evidence was synthesized narratively and thematically across the conceptual domains presented in Section 3, Section 4, Section 5, Section 6, Section 7 and Section 8.

## 3. Epidemiological Convergence Between Viral Hepatitis, HIV, and STI High-Risk Associated Populations

The epidemiology of viral hepatitis has changed substantially over the last two decades. Whereas HBV and HCV were historically conceptualized predominantly as blood-borne infections linked to transfusion or injection drug use, contemporary transmission dynamics overlap markedly with sexual health networks, particularly among MSM, individuals exposed to chemsex, HIV-positive populations, and PrEP users [1,8]. This convergence is dissolving the traditional boundaries between viral hepatitis programs and STI/HIV care pathways, with implications for both surveillance and clinical management around shared transmission ecosystems rather than isolated pathogens. This epidemiological convergence is not merely descriptive: it defines the populations and healthcare entry points around which the operational framework proposed in Section 8 is built.

### 3.1. Sexually Transmitted HCV Among MSM and Reinfection After Cure

The recognition of sexually transmitted HCV among MSM was a turning point in HCV epidemiology: pooled meta-analyses estimate HCV seroprevalence around 3.4% among MSM, substantially higher in HIV-positive than HIV-negative men [8]. Early-2000s European reports identified clusters of acute HCV in HIV-positive MSM without parenteral risk, linked to high-risk sexual practices, traumatic exposure, group sex, ulcerative STIs, and recreational drug use [12,47,48]; phylogenetic analyses subsequently confirmed large international transmission clusters across Europe and Australia [13,49,50]. Reinfection after sustained virological response emerged as a major challenge during the direct-acting antiviral era, particularly in HIV-positive MSM with ongoing high-risk behavior: cohort studies from Western Europe, Switzerland, the UK, Australia, and Spain report reinfection incidence that, in some settings, approaches or exceeds primary HCV incidence [16,17,51,52,53,54]. Globally, anti-HCV seroprevalence among people living with HIV is approximately 6.2% (around 2.3 million coinfected individuals), reaching up to 82% among PWID [18]; in Spain, active HCV prevalence in PLWH fell from 22.1% (2015) to 2.2% (2019) with broad DAA access [55]. These observations shift the conceptual model of HCV care from a linear, cure-oriented pathway toward longitudinal surveillance embedded within sexual health and addiction services.

### 3.2. PrEP and Chemsex: Extending the Convergence to HIV-Negative MSM

The expansion of HIV PrEP produced an additional epidemiological transition: cohorts in the Netherlands, France, Australia, and Spain reported HCV incidence among HIV-negative MSM on PrEP alongside elevated bacterial STIs and recurrent exposures [14,56]. Reported incidence ranged from approximately 0.9 to 2.0 per 100 person-years across these cohorts, generally declining over time in parallel with DAA scale-up [14,15,57,58]. Sexualised recreational drug use (chemsex) is one of the most consistent behavioral correlates of these infections, associated with condomless sex, multiple partners, traumatic practices, group sex, and STI acquisition [59,60,61], with further associations to poly-drug use and broader sexual-health risk documented in HIV-negative and HIV-positive MSM [38,62,63,64]. Chemsex environments combine behavioral vulnerability, mental health disorders, substance dependence, social marginalization, and barriers to healthcare access—conditions that may simultaneously amplify transmission and delay diagnosis [65,66].

### 3.3. HBV, HDV, and the Broader STI-Associated Risk Landscape

HBV epidemiology overlaps substantially with STI-associated populations: MSM remain a high-prevalence group despite vaccine availability, while migrants, incarcerated populations, sex workers, and socially marginalized individuals frequently present overlapping vulnerabilities involving HIV, STIs, and viral hepatitis, and STI clinics often represent one of their few points of healthcare contact [18,19,34,67,68]. HDV adds a further layer of complexity, since HDV requires HBV coinfection. Global HDV burden estimates vary substantially by population sampled and meta-analytic method: Stockdale et al. estimated pooled anti-HDV seroprevalence among HBsAg-positive individuals at approximately 4.5% (~12 million people worldwide) [21], versus a markedly higher 13% reported by Chen et al. [69], with the adjusted Polaris analysis of 25 countries attributing much of this divergence to sampling frame, assay performance, and study selection [22]. Regardless of the precise estimate, under-recognition is consistently most marked among migrants, HIV-positive individuals, and people who inject drugs, with pooled anti-HDV seroprevalence reaching approximately 8.4% in sub-Saharan Africa [70] and around 8% among tested US Veterans Affairs HBsAg-positive patients [71]—illustrating both under-diagnosis and the disproportionate burden of advanced liver disease, consistently above earlier estimates across sub-Saharan Africa, Asia, and Western systems [70,71,72].

### 3.4. Convergent Settings Beyond Sexual Health

**Figure 1:**
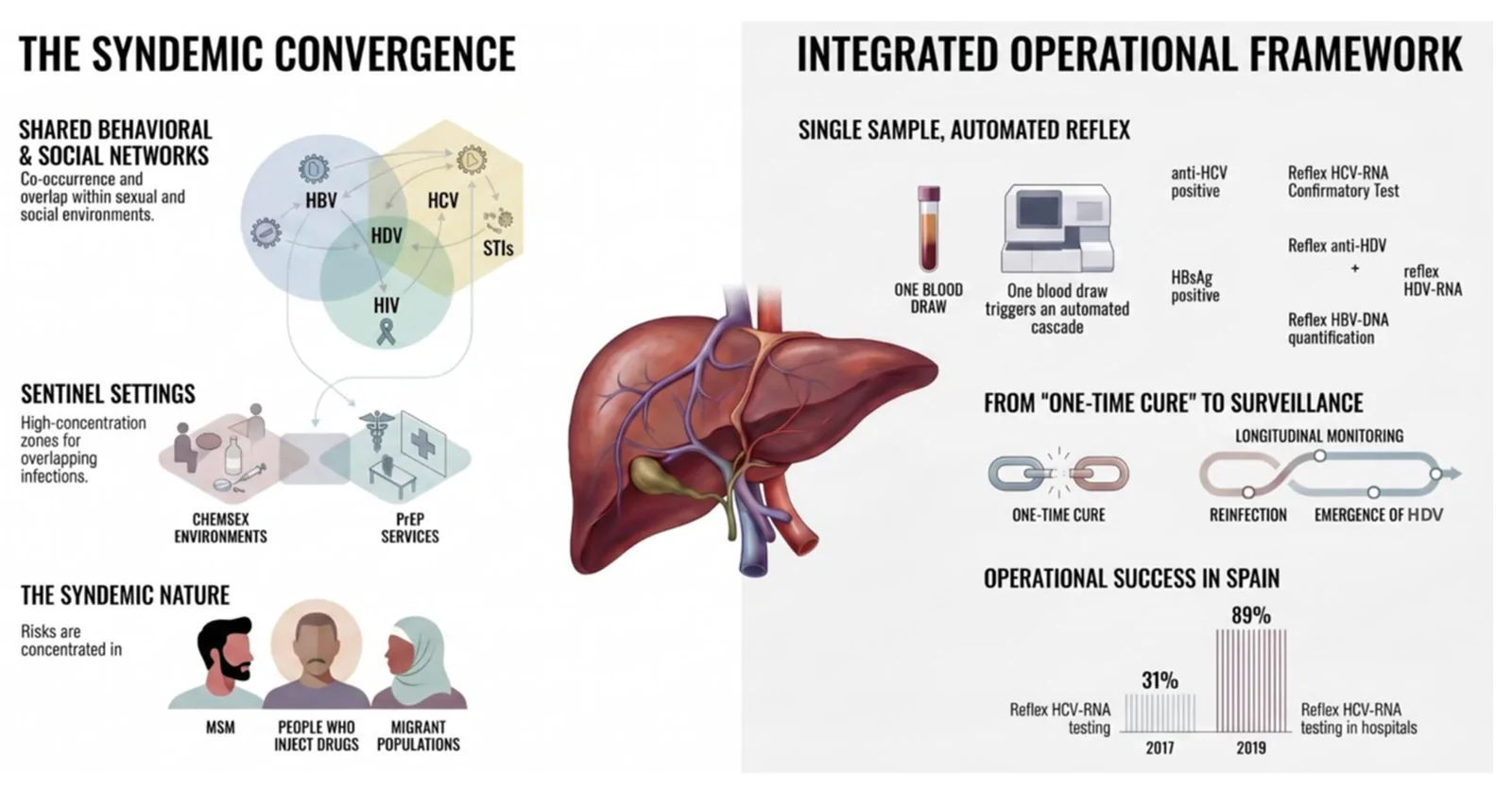
The syndemic convergence of viral hepatitis, HIV, and sexually transmitted infections and its translation into an integrated operational framework. The left panel (“The Syndemic Convergence”) depicts how HBV, HCV, HDV, HIV, and bacterial STIs cluster within shared behavioral, sexual, and social networks—with chemsex environments and PrEP services acting as sentinel settings that concentrate overlapping risk. The right panel (“Integrated Operational Framework”) shows how this convergence is operationalized at a single high-contact entry point: a single sample feeds an automated reflex cascade (e.g., reflex HCV-RNA after positive anti-HCV; reflex anti-HDV after positive HBsAg), with HDV serving as the structural test case for diagnostic fragmentation, and care shifting from one-time cure toward longitudinal surveillance of reinfection and emergence. The liver at the center links the epidemiological and operational halves, underscoring that integration is achieved through workflow redesign around the populations served rather than through new technology. HBsAg, hepatitis B surface antigen; HBV, hepatitis B virus; HCV, hepatitis C virus; HDV, hepatitis D virus; HIV, human immunodeficiency virus; PrEP, pre-exposure prophylaxis; STI, sexually transmitted infection. The ‘Operational success in Spain’ panel illustrates the proportion of Spanish hospitals performing reflex HCV-RNA testing, based on national multicentre implementation data collected in 2017 (31%) and 2019 (89%) [29,82,83,84,85].

Correctional facilities show disproportionately high prevalence of HCV, HBV, HIV, and substance use disorders, frequently combined with limited healthcare access and interrupted continuity of care [19,73,74]; globally, an estimated 10.2 million people pass through carceral settings each year, with pooled anti-HCV seroprevalence around 15.1%, HBsAg around 4.8%, and HIV around 3.8% [19]. In Spain, contemporary modeling estimates active HCV prevalence at 0.14% in the general population (≈54,500 individuals) but at 12.5% among current PWID and 8.4% among MSM engaging in chemsex, with around 29% of cases still undiagnosed [75]; in addition, the estimated number of anti-HCV-positive individuals among adult migrants exceeds 100,000, with concentration in specific autonomous communities and origin countries [20]. Similar overlaps are documented among trans women, sex workers, homeless populations, and migrants, where structural barriers contribute more strongly to ongoing transmission than purely biological factors [76,77,78,79,80]. These epidemiological transitions carry direct implications for healthcare organization: disease-specific models built around separate HIV, hepatitis, STI, and addiction services no longer reflect contemporary transmission networks, and STI clinics, HIV programs, PrEP services, addiction centers, emergency departments, prison healthcare systems, and community-based screening initiatives represent critical opportunities for combined viral hepatitis diagnosis [29,30,81]. The epidemiological and operational implications of this syndemic convergence are summarized in Figure 1.

## 4. Integrated Diagnostic Models and Enabling Technologies

Combined diagnostic strategies reorganize screening around shared epidemiological risk rather than isolated pathogens. The technologies reviewed below are best understood not as ends in themselves but as the building blocks of the implementation strategy developed in Section 8. Within this framework, individuals exposed to one STI-associated risk factor are evaluated for multiple infections through coordinated pathways combining serological testing, reflex molecular confirmation, electronic integration, and rapid linkage-to-care) [86,87,88]. This section synthesizes the rationale and the principal enabling technologies; their operational integration is detailed in the practical framework presented in Section 8 and summarized in Table 1, Table 2 and Table 3.

### 4.1. Rationale: Shared Exposure Ecosystems and Missed-Opportunity Testing

MSM, HIV-positive individuals, PrEP users, people who inject drugs, incarcerated populations, migrants from endemic regions, and individuals participating in chemsex frequently present simultaneous or sequential exposure to HIV, HBV, HCV, HDV, and bacterial STIs [9,14,15]. Testing for only one pathogen during a healthcare contact often represents a missed opportunity for prevention and early diagnosis. In addition, STI services provide an important opportunity to assess hepatitis A susceptibility and offer vaccination to individuals at ongoing sexual risk, particularly MSM, where hepatitis A is increasingly recognized as a vaccine-preventable sexually transmitted infection [91,128]. Opt-out HIV testing models implemented in emergency departments and sexual health clinics have improved identification of previously undiagnosed HIV while creating opportunities for HBV and HCV screening [92,93]. Combined viral hepatitis screening programs embedded within STI services, HIV clinics, or emergency departments have identified substantial numbers of previously unrecognized HBV and HCV infections, with prevalence among unselected attendees often several-fold higher than in the general population. Representative emergency-department experiences illustrate this yield: opt-out panel testing in Ireland identified HIV in 0.2%, HBsAg in 0.9%, and anti-HCV in 4.0% of 5003 patients [94]; US ED programs found anti-HCV positivity of 9.7–13%, with substantial confirmed viraemia [95,96,102]; and published implementation analyses show that, even in well-resourced settings, concomitant hepatitis testing during STI evaluation is frequently incomplete, with the lowest concordant testing among those evaluated only by genital swab [30,95,103]. European, Irish, and US implementation studies in STI services, PrEP clinics, and pregnancy care similarly demonstrate substantial diagnostic yield [92,104,105,106].

### 4.2. Reflex Testing

Reflex testing constitutes the operational core of contemporary multi-pathogen diagnostic pathways. In reflex HCV-RNA testing, confirmatory molecular assays are performed automatically following positive serology using the same sample, eliminating the need for an additional healthcare encounter. Studies in urban healthcare systems, addiction services, prison settings, and STI clinics report higher rates of diagnostic completion and earlier treatment initiation when reflex testing is implemented compared with standard multi-step algorithms [24,94,129,130]. Spanish national multicenter implementation experience has documented improvements in cascade completion and time-to-treatment after the introduction of reflex HCV-RNA: between 2017 and 2019, the proportion of Spanish hospitals performing reflex HCV-RNA testing rose from 31% to 89% following coordinated guidance from scientific societies, with parallel growth of automated alerts to specialist physicians [29,82,83,84,85]. Similar principles apply to HBV and HDV diagnostic algorithms, particularly through automated reflex anti-HDV testing in HBsAg-positive individuals; several European laboratory networks have reported substantial increases in HDV testing coverage after implementation [119,120,121]. The Spanish Andalusia cohort additionally demonstrated that double-reflex HDV testing (anti-HDV in HBsAg-positive samples and HDV-RNA in anti-HDV-positive samples) is both clinically and economically efficient [122].

### 4.3. Multiplex Platforms, Point-of-Care Diagnostics, and DBS

Multiplex serological or molecular platforms allow simultaneous testing for HIV, HBV, HCV, and syphilis, reducing logistical complexity and patient burden. They appear particularly useful in community-based and outreach interventions where repeated sampling is difficult [131,132,133,134]. Multiplex bead-based and quadruple POC assays for HIV, HBV, HCV, and syphilis are in active development and validation [135,136,137]. Point-of-care (POC) platforms capable of near-patient HCV-RNA detection from finger-stick or capillary blood allow same-day confirmation of active infection: the Xpert HCV Viral Load assay using finger-stick capillary samples has shown sensitivity around 98% and specificity around 95%, with results available in approximately one hour [25,138]; HCV core antigen testing offers an economical alternative with pooled sensitivity around 93% and specificity around 98% versus HCV-RNA [139]. The MINMON trial confirmed that minimal-monitoring DAA strategies based on simplified diagnostics may achieve high SVR rates without intensive laboratory infrastructure [140]. Dried blood spot (DBS) testing complements these strategies by enabling capillary sampling on absorbent filter paper without cold-chain requirements; its diagnostic performance for HCV-RNA and HBV-DNA quantification is acceptable for screening purposes, particularly in prisons, harm-reduction services, and outreach programs [141,142,143,144]. The summary characteristics of each technology are presented in Table 2. Dried blood spot (DBS) and plasma separation card (PSC) samples, although both paper-based, are not analytically interchangeable and require format-specific validation; similarly, HDV-RNA quantification from DBS currently lacks standardized, harmonized assay protocols, and cross-reactivity between HBsAg/anti-HBc/anti-HBs and anti-HDV serologies should be considered when interpreting combined panels.

### 4.4. Digital Integration: EHR Alerts and Emerging Analytics

Digital integration also extends to EHR-based alerts and automated screening reminders, which have been associated with improved HCV screening uptake and treatment initiation in primary care and emergency departments [26,99,100,101]. Artificial intelligence and machine learning may extend these capabilities: for example, a machine-learning model applied to electronic health record data has been used to flag patients with a high likelihood of undiagnosed hepatitis C virus infection for targeted testing [145], illustrating a concrete, if still limited, application beyond the broader case-finding literature [146,147,148]. However, these tools also raise concerns about data quality, algorithmic bias, and the risk of reinforcing existing healthcare inequities, and should be deployed with explicit attention to representativeness and equity [123,124,125,126,127].

### 4.5. Implementation Barriers

Despite the maturity of these technologies, implementation remains heterogeneous. Even in high-income systems, reflex testing is incompletely adopted, HDV screening is inconsistently performed, and interoperable electronic pathways are frequently absent [7,81,149]; recent Spanish national tracking data show that, between 2022 and 2024, the proportion of hospitals performing reflex testing for HBV increased by 33%, for HDV by 45%, for dual HBV–HDV by 79%, anti-HDV testing by 18%, and HDV-RNA testing by 21%, alongside automated alerts to specialists rising 24% for HBV and 53% for HDV [97]. The remaining barriers are largely operational—separated funding structures, limited interoperability, insufficient cross-specialty coordination, provider unfamiliarity, persistent stigma, and unaligned reimbursement [30,98]. Section 5, Section 6, Section 7 and Section 8 develop the components of a combined response and Section 8 proposes a practical, STI-clinic-centered framework. Table 3 summarizes barriers and proposed solutions.

## 5. Linkage-to-Care, Retention, and Longitudinal Surveillance

Diagnosis alone is not sufficient for viral hepatitis elimination. Across HIV, HBV, HCV, and HDV care pathways, the ability to maintain continuity between screening, confirmatory diagnosis, treatment initiation, follow-up, and long-term surveillance is a major determinant of clinical and public health outcomes. Major losses have historically occurred at every stage of this cascade, particularly among vulnerable populations characterized by unstable healthcare engagement [150,151].

### 5.1. Simplified Pathways, EHR Alerts, and Patient Navigation

Simplified pathways combining reflex molecular testing, same-day confirmatory diagnosis, decentralized assessment, and rapid treatment initiation have been associated with reduced attrition compared with conventional multistep referral systems [24,29,30]. EHR-based alerts and best-practice notifications have been associated with increased HCV screening and curative treatment, particularly in primary care and emergency department settings. Automated EHR-triggered best-practice alerts have been associated with high rates of linkage-to-care and short times to first specialist appointment for newly diagnosed HCV viraemia and HIV [26,99]. Patient navigation models likewise show benefit in populations affected by social vulnerability or previous healthcare disengagement [100,152,153,154]. Peer-support interventions involving individuals with lived experience of injection drug use, incarceration, HIV, or hepatitis treatment may further facilitate engagement through trust-building and continuity during transitions between community and institutional settings [89,90,95,155].

### 5.2. Decentralization and Telemedicine

Decentralization is another component of effective linkage-to-care: hospital-based hepatology models often create access barriers for populations with unstable housing, active substance use, psychiatric illness, or limited mobility, whereas community-based testing, addiction-centered care, prison-integrated treatment, mobile outreach, and STI-clinic-centered management may improve retention [27,107,108]. Telemedicine has further enabled decentralized HCV care with SVR rates comparable to, or exceeding, specialist-centered outcomes: the seminal ECHO model showed equivalence between telementored primary care (58.2%) and hepatology centers (57.5%) [156]; the MINMON trial achieved 95% SVR with minimal in-person monitoring [140]; randomized trials of telemedicine-based HCV–opioid-use-disorder care and simplified PWID models achieved SVR above 90% [28,31]; and a Spanish penitentiary telemedicine program produced 30.6% cost savings while maintaining 94.7–100% SVR [157].

### 5.3. Beyond Cure: Longitudinal Surveillance and Reinfection Monitoring

Elimination-oriented care increasingly requires moving beyond cure as the endpoint. In populations with persistent exposure risk (MSM engaged in chemsex, PrEP users, people who inject drugs), reinfection after successful HCV treatment has become a major challenge, with reported rates of 1.2–7.3 per 100 person-years across Spanish, European, Dutch, and Swiss cohorts [16,17,51,52,158,159]. PrEP infrastructures, with structured periodic STI testing, create natural opportunities for longitudinal hepatitis surveillance, including recurrent HCV screening, HBV vaccination assessment, and reinfection monitoring [14,34,160].

## 6. HDV as the Stress Test of Diagnostic Fragmentation

Among all chronic viral hepatitis infections, HDV may best illustrate the consequences of fragmented diagnostic systems, remaining profoundly under-diagnosed even in high-income healthcare systems with advanced laboratory infrastructure [21,22,70].

### 6.1. Selective Testing Has Proven Insufficient

HDV’s dependence on HBsAg led many clinicians to conceptualize it as a rare HBV complication rather than an entity requiring systematic evaluation, so testing has typically been reserved for selected patients rather than incorporated into routine HBsAg-positive algorithms [161,162,163]. Contemporary studies suggest HDV prevalence has been consistently underestimated, particularly among migrants, MSM, people who inject drugs, incarcerated populations, and individuals with HIV coinfection [7,21,22].

### 6.2. The Implementation Gap

Under-diagnosis of HDV is not primarily a technological problem: serological and molecular tests are widely available, but HDV integration within routine HBV care pathways remains incomplete—HBsAg positivity does not automatically trigger anti-HDV testing, and reflex algorithms remain absent. HDV testing therefore depends on individual clinician awareness, institutional culture, or specialist expertise, generating heterogeneity across centers [7,119,120,164].

### 6.3. Clinical Consequences and the Bulevirtide Era

This fragmentation matters because HDV substantially accelerates disease progression, with faster fibrosis and higher risks of cirrhosis, decompensation, hepatocellular carcinoma, and liver-related mortality than HBV monoinfection [21,165,166]. The recent availability of bulevirtide has transformed a previously limited therapeutic landscape: the phase 3 MYR301 trial demonstrated a markedly superior combined biochemical–virological response with bulevirtide compared with delayed treatment, with a favorable tolerability profile confirmed in integrated safety analyses [23,167,168,169]. Real-world European cohorts, including the Italian D-SHIELD programme, confirm consistent responses, including in compensated cirrhosis, and combination strategies with pegylated interferon and tenofovir continue to be explored [170,171,172,173,174,175].

### 6.4. HDV and the Limits of Pathogen-Centered Care

Many individuals at risk for HDV also have elevated prevalence of HIV, HCV, STIs, migration-related vulnerability, incarceration history, or substance use, yet healthcare systems often continue to manage HBV/HDV separately from STI, HIV, addiction, and community-based screening services [7,73,81,176,177]. Migration-associated fragmentation deserves particular attention: low-threshold combined screening within community, migrant-health, or sexual-health programs may outperform specialist-centered referral [76,78]. Box 2 summarizes the operational case for combined HDV diagnosis.

Box 2 **HDV—the stress test of diagnostic fragmentation.**

- Underestimated burden. Recent meta-analyses estimate higher HDV seroprevalence than historically assumed, with marked under-recognition among migrants, HIV-positive individuals, and people who inject drugs.
- Selective testing has failed. Risk-based strategies miss a substantial proportion of cases; systematic anti-HDV testing in all HBsAg-positive individuals is needed.
- Implementation, not technology, is the bottleneck. Reliable assays exist; the persistent gap is the absence of automated reflex algorithms in routine HBV care.
- Clinical stakes are high. HDV accelerates fibrosis, cirrhosis, decompensation, hepatocellular carcinoma, and mortality—and the bulevirtide era makes timely diagnosis clinically actionable.
- Operational solution. Mandatory reflex anti-HDV in every HBsAg-positive sample, with reflex HDV-RNA on positive samples, integrated within STI, HIV, prison, addiction, and migrant-health services.

(Authors’ synthesis).

## 7. Microelimination and Implementation Experience

Translating effective antivirals into population-level elimination requires diagnostic coverage, linkage-to-care, retention, surveillance, and sustained implementation capacity [6,86,87].

### 7.1. The Microelimination Framework

Rather than relying exclusively on broad national strategies, microelimination focuses on defined subpopulations, healthcare environments, or transmission networks where elimination appears realistically achievable through targeted interventions [178,179,180]. Region-specific syntheses such as the recent Latin-American analysis of barriers to HCV screening illustrate how this framework may be adapted to fragmented health systems and key vulnerable populations [181]; real-world programs integrating HCV care into addiction, mental-health, or marginally housed populations have achieved SVR rates of 90–95%, comparable to those observed in less complex populations when administrative and clinical barriers are removed [89]. High-risk populations concentrate a substantial proportion of ongoing transmission and undiagnosed infection; many already interact regularly with identifiable healthcare structures (STI clinics, HIV programs, prisons, addiction services, migrant health programs, community outreach), making them tractable targets for combined interventions.

### 7.2. Sexual Health, HIV/PrEP, Prisons, and PWID as Platforms

STI clinics and HIV services manage populations characterized by recurrent exposure, frequent healthcare contact, elevated STI prevalence, and established infrastructures for periodic screening, so integration of HBV, HCV, and HDV diagnosis into these platforms aligns with existing workflows [34,68,104]. PrEP infrastructures appear particularly attractive for longitudinal hepatitis surveillance [14,159], and HIV-cohort microelimination experience in Switzerland, France, and the United Kingdom suggests the feasibility of HCV elimination targets in HIV-positive MSM through coordinated screening, treatment scale-up, and reinfection surveillance [182,183,184].

Prison systems show disproportionately high prevalence of HCV, HBV, HIV, and substance use, though incarceration may paradoxically facilitate healthcare access: opt-out combined screening, decentralized treatment, and coordinated post-release continuity have been associated with reduced viral hepatitis burden across several countries, illustrated by Spain’s Cantabria microelimination program [107,108,109,110], a navigator-assisted test-and-treat strategy achieving 92.8% screening coverage [111], telemedicine-supported management with 30.6% cost savings [157], and JAILFREE-C cost-effectiveness modeling supporting nationwide DAA scale-up [112,113], with comparable experiences in Italian, English, and US correctional systems [114,115,116,117,118]. People who inject drugs constitute another major microelimination priority: harm-reduction environments, opioid substitution programs, mobile clinics, and community outreach provide platforms for combined testing and treatment with simplified diagnostics and decentralized antiviral access [27,185,186], as illustrated by pilot programs in Hong Kong, Pakistan, and the Cherokee Nation experience in the United States [187,188,189].

### 7.3. Cost-Effectiveness and Adaptive System Redesign

Cost-effectiveness analyses from Spanish, English, Italian, and US healthcare systems consistently report combined screening and reflex pathways as cost-effective, and in some scenarios cost-saving, at conventional willingness-to-pay thresholds [30,190,191,192], while global investment-case modeling similarly supports scaled combined screening and treatment, including treatment-as-prevention in key populations [193,194,195,196]. Implementation barriers are highly context-dependent, however: strategies successful in tertiary centers may fail in decentralized or low-resource settings, and interventions effective in stable populations may prove inadequate among socially vulnerable groups, so elimination strategies require adaptive rather than uniform models [37,88]. Digital infrastructures may facilitate implementation at scale while introducing ethical considerations regarding confidentiality, algorithmic bias, and digital exclusion of marginalized populations [28,101,123].

## 8. A Practical Framework for STI-Based Integrated Viral Hepatitis Diagnosis

**Figure 2:**
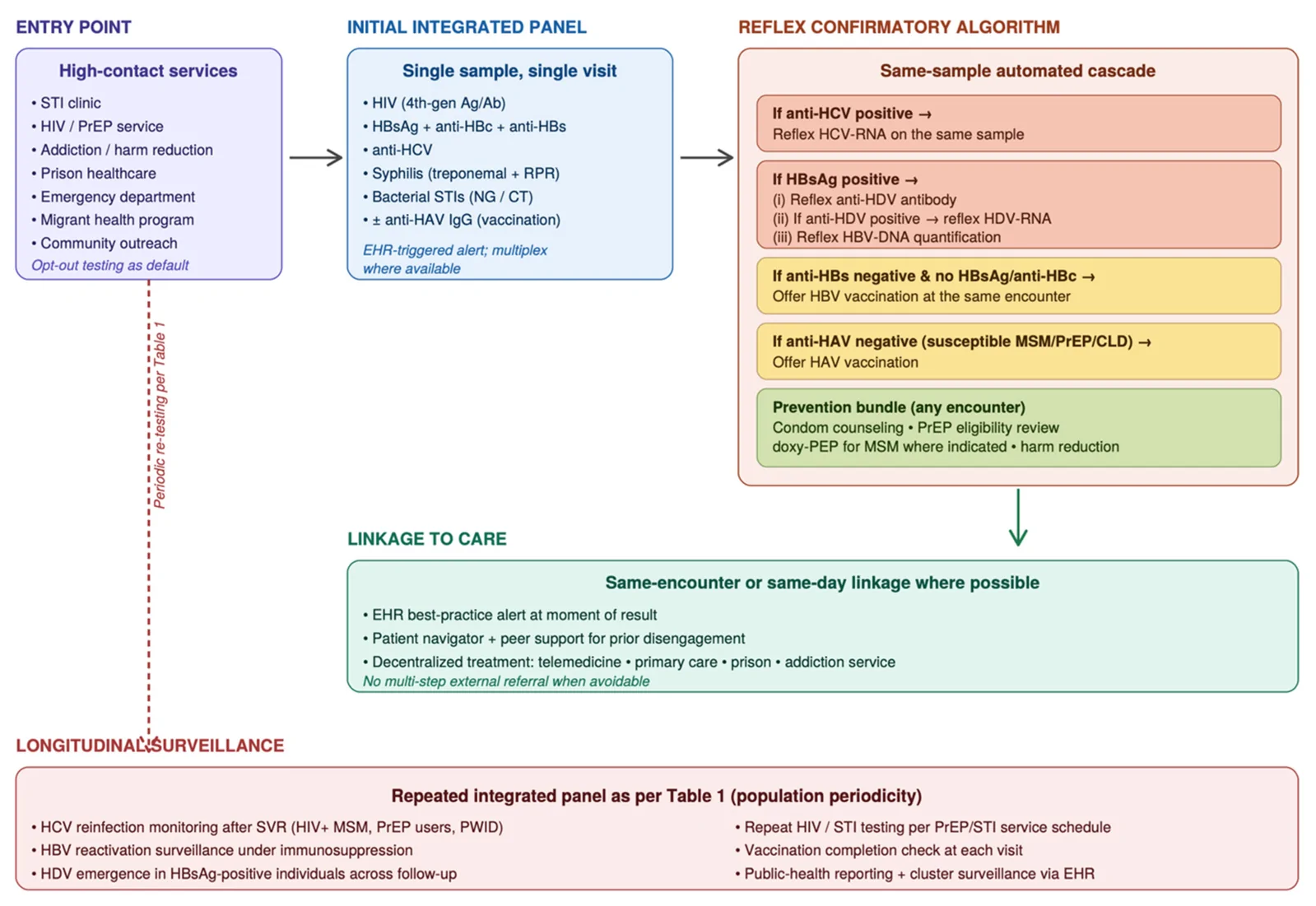
Operational framework for STI-clinic-centered viral hepatitis diagnosis. Integrated diagnostic pathway synthesizing the evidence reviewed in this manuscript, illustrating how STI services, HIV/PrEP programs, addiction care, prison healthcare, emergency departments, and community-based screening initiatives may serve as common entry points for coordinated HBV, HCV, HDV, HIV, and STI diagnosis, prevention, linkage-to-care, and longitudinal surveillance.

Building on the syndemic and operational framework illustrated in Figure 1, we propose a practical STI-clinic-centered model for integrated viral hepatitis diagnosis. The preceding sections describe a converging epidemiology, a mature diagnostic toolkit, and substantial implementation gaps. We propose—as authors, not as a consensus or standard—a practical, STI-clinic-centered framework that may help translate this evidence into routine clinical workflow, integrating reflex diagnostics, periodicity by population, multidisciplinary linkage, and longitudinal surveillance, and broadly aligned with current WHO, EASL, EACS, and AASLD–IDSA recommendations [31,32,33,44]. This framework has not been prospectively evaluated as a combined package, and its real-world effectiveness, cost-effectiveness, and implementation may vary across healthcare systems; see Section 10 for a fuller discussion of these limitations. The framework is summarized in Figure 2 and Table 1, Table 2 and Table 3, with the principal recommendations distilled in Box 3.

Box 3 **Practical recommendations for STI-clinic-centered integrated viral hepatitis diagnosis.**

- Entry panel. Consider offering HIV (4th-gen Ag/Ab), HBsAg + anti-HBc + anti-HBs, anti-HCV, syphilis (treponemal + RPR), and bacterial STIs (NG/CT) at first contact in any STI, HIV/PrEP, addiction, prison, ED, migrant health, or community outreach service, on an opt-out basis where feasible.
- Reflex confirmation. Configure laboratory-side automated reflex algorithms: HCV-RNA after positive anti-HCV; anti-HDV after positive HBsAg; HDV-RNA after positive anti-HDV; HBV-DNA in newly diagnosed HBsAg-positive individuals—all on the same specimen.
- Vaccinate at point of care. Offer HBV vaccination to susceptible individuals (anti-HBs–negative without HBsAg or anti-HBc) at the same encounter; offer HAV vaccination to susceptible MSM, PrEP users, and individuals with chronic liver disease.
- Prevention bundle. Combine condom counseling, harm reduction, opioid substitution where indicated, PrEP eligibility review, and doxy-PEP for eligible individuals, integrated into the same visit.
- Periodicity by population (Table 1). 3-monthly testing in PrEP users; 3–6-monthly in HIV-positive MSM and active chemsex networks; 6–12-monthly in PWID; entry + annual + release in incarcerated populations; baseline in migrants from endemic regions.
- Same-encounter linkage. Use EHR alerts at the moment of result; deploy patient navigators and peer support for prior disengagement; avoid multi-step external referral whenever possible.
- Decentralize treatment. Enable HCV/HBV/HDV treatment within primary care, addiction services, prison healthcare, and via telemedicine, rather than restricting to hospital hepatology.
- Mandatory institutional reflex anti-HDV. Anti-HDV in every HBsAg-positive sample is the single most impactful change available to most laboratories without additional infrastructure.
- Longitudinal surveillance. Repeat the integrated panel per Table 1 across follow-up to detect HCV reinfection, HBV reactivation, HDV emergence, and incident HIV/STIs.
- Bundled reimbursement and EHR interoperability. Align financial structures with multi-pathogen panels; implement shared EHR alerts across STI, HIV, hepatology, ID, primary care, and microbiology services.

### 8.1. Entry Point and Initial Integrated Panel

Any individual presenting to an STI service, HIV/PrEP service, addiction or harm-reduction program, prison healthcare unit, emergency department, or community-based outreach platform with at least one STI-associated risk factor may be offered an initial combined panel comprising: HIV (fourth-generation antigen/antibody assay), HBsAg (with anti-HBc and anti-HBs to assess immunity status), anti-HCV antibody, and syphilis (treponemal and non-treponemal tests). Where epidemiologically indicated, anti-HAV IgG should be obtained to guide vaccination [33,34,44,68]. Where feasible, opt-out testing has been associated with improvements in uptake and yield [92,93].

### 8.2. Reflex Confirmatory and Complementary Testing

The initial panel may be coupled with automated laboratory algorithms generating reflex confirmatory tests on the same specimen: (i) reflex HCV-RNA testing if anti-HCV is positive; (ii) reflex anti-HDV testing if HBsAg is positive; (iii) reflex HDV-RNA testing if anti-HDV is positive; and (iv) reflex HBV-DNA in newly diagnosed HBsAg-positive individuals. This double- (and where feasible triple-) reflex strategy collapses what is conventionally a multi-visit cascade into a single sample, with consistently demonstrated improvements in cascade completion, time to treatment, and HDV detection [24,29,82,119,120,122].

### 8.3. Vaccination, Prevention, and Harm Reduction at Point of Care

Susceptible individuals (anti-HBs–negative without HBsAg or anti-HBc) may be offered HBV vaccination at the same encounter, in line with the 2022 ACIP recommendation of universal HBV vaccination for adults aged 19–59 years and risk-based vaccination for those aged 60 years or older now adopted in the United States and similar policies under discussion in several other high-income settings [43,197]. HAV vaccination may be offered to susceptible MSM, PrEP users, and individuals with chronic liver disease per guideline recommendations. Concomitant prevention should include condom counseling, needle-and-syringe program access, opioid substitution where indicated, behavioral chemsex support, and—for eligible individuals—initiation or reinforcement of HIV PrEP and consideration of doxycycline post-exposure prophylaxis (doxy-PEP) for bacterial STI prevention according to current guidance—phase 3 evidence shows approximately 65% reduction in overall bacterial STI incidence and reductions in more than 70% in syphilis and chlamydia and approximately 50% in gonorrhea in target populations [68,198,199].

### 8.4. Population-Specific Testing Periodicity

Once the entry panel and reflex algorithms are in place, periodic re-testing may be aligned with the epidemiological risk of each population. Table 1 summarizes proposed periodicity by population, drawing on current guidelines and cohort evidence. In brief: HIV-positive MSM and HIV-negative MSM on PrEP warrant 3- to 6-monthly screening for HIV, HCV-RNA, and bacterial STIs, with annual HBV serology if susceptible [14,33,34]. Individuals with active or recent injection drug use, residents of correctional facilities with extended sentences, and individuals in active chemsex networks similarly benefit from frequent re-testing aligned with their healthcare contact pattern [65,107,108]. Migrants from HBV/HDV-endemic regions warrant at least one comprehensive baseline combined panel, with HDV reflex testing in any HBsAg-positive individual.

### 8.5. Linkage, Treatment Access, and Longitudinal Surveillance

The diagnostic pathway may be coupled with explicit linkage architecture. Three operational principles emerge from the literature: (i) same-day or same-encounter linkage wherever possible, supported by EHR alerts and best-practice notifications [26,99]; (ii) navigator-assisted referral and peer support for individuals with prior healthcare disengagement [89,152,153]; and (iii) decentralized treatment delivery—including telemedicine and primary-care-, prison-, or addiction-service-based care—to reduce the geographic and institutional friction of conventional referral pathways [27,28,31]. Longitudinal surveillance—for HCV reinfection, HBV reactivation under immunosuppression, HDV emergence in HBsAg-positive individuals, and STI/HIV recurrence—may be routine within PrEP and STI services rather than an exception.

### 8.6. Anticipating Barriers

Implementation of this framework will encounter recurrent barriers across laboratory, financial, organizational, social, and population-specific dimensions. These barriers and proposed solutions are summarized in Table 3. Three deserve particular emphasis: (i) the laboratory-system requirement to validate, configure, and audit reflex algorithms—particularly for HDV, where they remain rare in many institutions [7]; (ii) reimbursement structures that need to align with bundled multi-pathogen testing rather than per-test billing [30,98]; and (iii) explicit integration of the framework into existing STI clinic, HIV/PrEP, prison, and addiction-service workflows, rather than as a parallel hepatology-led pathway, recognizing that the operational reality of integration is the redesign of routine clinical workflow rather than the deployment of additional infrastructure [81,86]. The cost-effectiveness evidence underpinning this framework [30,188,189,190,191,192,193,194] derives from heterogeneous healthcare systems, currencies, and willingness-to-pay thresholds, and predominantly models single-pathogen rather than fully integrated multi-pathogen screening pathways; formal economic evaluation of the combined framework proposed here has not been undertaken and represents a priority for future implementation research.

The applicability of this framework to low- and middle-income settings, where reflex molecular testing, laboratory information systems, and EHR infrastructure may be limited or unavailable, warrants further evaluation. As illustrated by implementation-barrier analyses from Latin America [179], adaptation in such settings may depend more heavily on simplified diagnostics (point-of-care assays, dried blood spots) and community-based delivery than on the laboratory-intensive reflex algorithms emphasized throughout this framework.

## 9. Future Perspectives

Future progress in combined viral hepatitis diagnosis depends less on new technologies than on integrating those already validated. Three priorities follow from the evidence reviewed here. First, HDV diagnosis is a tractable test case: reflex anti-HDV and reflex HDV-RNA testing are technically trivial, and Spanish implementation tracking shows this gap can close quickly once scientific societies, microbiology services, and hepatology coordinate around a shared algorithm [7,97,119,122]. Second, the diagnostic and confirmatory technologies discussed throughout this review—reflex molecular testing, dried blood spots, point-of-care assays, simplified treatment monitoring—are already in routine use somewhere; the open question is how to embed them in the workflows of the STI, HIV, and addiction services where high-risk individuals already attend [87,93,96,141]. Third, universal adult HBV vaccination becomes operationally meaningful only if anti-HBs and anti-HBc determination is built into the same encounter that triggers HCV and HIV testing [197]. Predictive analytics applied to EHR data may eventually contribute to case-finding [146] but should not displace these operational priorities, and an effective HCV vaccine, currently in early-phase development, would alter this landscape over a longer horizon [200].

## 10. Limitations

This review has several limitations that should be acknowledged. First, as a narrative rather than systematic review, it does not include a quantitative meta-analysis or formal risk-of-bias assessment of individual studies, and conclusions therefore reflect a thematic synthesis rather than pooled effect estimates. Study selection and synthesis were conducted by two authors (see authors’ contributions) on the basis of shared expertise and consensus, without a formal multi-reviewer disagreement-resolution protocol, consistent with the narrative rather than systematic design of this review.

Second, the available evidence is heterogeneous in design, populations, definitions, and outcomes. The concept of “combined screening” itself varies substantially across studies, encompassing simultaneous multi-pathogen testing, sequential opt-out programs, reflex laboratory algorithms, and broader healthcare-system redesign. This heterogeneity limits direct comparison between programs and may complicate transferability of specific findings to other healthcare contexts.

Third, the evidence base is dominated by studies from high-income countries with universal or near-universal healthcare coverage. Findings from these settings may not translate directly to low- and middle-income contexts, where infrastructure constraints, financing models, workforce availability, and regulatory environments differ substantially. The generalizability of the proposed framework to such settings warrants further evaluation.

Fourth, much of the cited literature consists of observational cohorts, implementation studies, and cost-effectiveness analyses rather than randomized controlled trials. While the consistency of findings across diverse settings strengthens confidence in the overall direction of effects, residual confounding, selection bias, and publication bias toward positive implementation outcomes cannot be excluded.

Fifth, the proposed operational framework (Section 8; Figure 2; Table 1, Table 2 and Table 3) is grounded in published evidence and current guidelines but has not itself been formally evaluated as a combined package. Its real-world effectiveness, acceptability, and cost-effectiveness in different healthcare systems will require prospective implementation studies. The recommended testing periodicity ranges represent reasonable syntheses of current guidelines and cohort evidence; they should be adapted to local epidemiology, clinical judgment, and individual risk profiles.

Finally, the field is evolving rapidly. New diagnostic technologies, therapeutic agents (notably for HDV), vaccination strategies, and implementation experiences are emerging continuously. The framework proposed here should therefore be regarded as a starting point that will require iterative refinement as the evidence base matures.

## 11. Conclusions

Viral hepatitis epidemiology has changed substantially over two decades. HBV, HCV, HDV, HIV, and bacterial STIs increasingly converge within overlapping behavioral, social, and healthcare networks. This convergence challenges traditional pathogen-specific diagnostic models and exposes the limitations of fragmented healthcare systems. What integrated diagnosis really requires is workflow redesign, not new technology. Reflex testing, multiplex platforms, point-of-care assays, dried blood spots, EHR alerts, decentralized pathways, and longitudinal surveillance are all in routine use somewhere; the open question is how to combine them within STI, HIV, and addiction service workflows. Many of the necessary technologies already exist; the principal barriers reside in implementation, interoperability, and unequal healthcare access.

HCV elimination efforts illustrate both the potential and the limitations of contemporary antiviral medicine. Highly effective therapies are not enough by themselves when diagnosis, linkage-to-care, retention, and reinfection surveillance remain incomplete. Similar challenges are evident in HBV and particularly HDV—the latter representing perhaps the clearest expression of the diagnostic gaps generated by fragmented healthcare architectures, and a useful indicator of broader elimination capacity. Microelimination strategies provide a pragmatic framework for translating elimination goals into operational interventions; STI clinics, HIV programs, prisons, harm-reduction environments, and PrEP infrastructures may be particularly valuable platforms because they combine concentrated epidemiological burden with opportunities for repeated healthcare contact and longitudinal surveillance.

The practical, STI-clinic-centered framework proposed in this review (Figure 2; Table 1, Table 2 and Table 3; Box 3) is intended to translate this evidence into routine clinical workflow. Implementation depends less on new diagnostic tools than on the willingness of healthcare systems to redesign workflows around the populations they serve.

## Figures and Tables

**Table 1:** Proposed testing periodicity for HBV, HCV, HDV, and HIV by STI-associated population.

Population	Dominant Risk	Infections to Screen	Suggested Periodicity	Preferred Technology	Evidence Basis	Source(s)
HIV-positive MSM	Sexual + chemsex	HCV-RNA, syphilis, NG/CT, HBV serology if susceptible	3–6 months	Reflex HCV-RNA; multiplex STI	Guideline-aligned + cohort evidence	EACS v12.0 [33]; CDC STI Guidelines 2021 [34]; Hoornenborg et al. PrEP cohort [14]
HIV-negative MSM on PrEP	Sexual + chemsex	HIV, HCV-RNA, syphilis, NG/CT, HBV serology if susceptible	3 months	Reflex HCV-RNA; doxy-PEP eligibility review	Guideline-aligned + cohort evidence	EACS v12.0 [33]; CDC STI Guidelines 2021 [34]; Hoornenborg et al. [14]
Active chemsex networks	Sexual + drug use	HIV, HCV-RNA, HBsAg, syphilis, NG/CT	3–6 months	Multiplex POC where available; behavioral support	Cohort/implementation evidence	Lazarus et al. [65]; Cuadrado et al., Alonso-Peña et al. [89,90]
People who inject drugs	Parenteral + sexual	HIV, HBsAg + reflex anti-HDV, HCV-RNA, syphilis	6–12 months	DBS/POC; harm reduction integration	Cohort/implementation evidence	refs. [65,89,90]
Incarcerated populations	Mixed structural	HIV, HBsAg + reflex anti-HDV, anti-HCV reflex RNA, syphilis	Entry + annual; release	Opt-out integrated panel; DBS feasible	Cohort evidence (entry/annual) + authors’ synthesis (release timing)	refs. [89,90]; release-testing interval not guideline-cited
Migrants from endemic regions	Endemic exposure	HBsAg + reflex anti-HDV, anti-HCV reflex RNA, HIV, syphilis	Baseline; risk-based repeat	Low-threshold community/migrant-health programs	Authors’ synthesis	
Sex workers	Sexual	HIV, HBsAg, anti-HCV reflex RNA, syphilis, NG/CT	3–6 months	Multiplex; community outreach	Authors’ synthesis (general STI guidance)	CDC STI Guidelines 2021 [34] (general, not periodicity-specific)
ED attendees in high-prevalence catchments	Mixed/unknown	HIV opt-out, HBsAg, anti-HCV reflex RNA	Per attendance	EHR-triggered opt-out + reflex panel	Cohort/implementation evidence	Opt-out ED studies [91,92,93,94,95,96]

Abbreviations: HIV, human immunodeficiency Virus; HCV, Hepatitis C Virus; HBV, Hepatitis B Virus; HBsAg, Hepatitis B S Antigen; CT, chlamydia trachomatis; DBS, dried blood spot; doxy-PEP, doxycycline post-exposure prophylaxis; ED, emergency department; EHR, electronic health record; MSM, men who have sex with men; NG, Neisseria gonorrhoeae; POC, point of care; PrEP, pre-exposure prophylaxis. Periodicity ranges synthesize current guideline recommendations and cohort evidence; clinical judgment should adapt them to the individual case. Evidence basis: ‘Guideline-aligned’ indicates alignment with an explicit recommendation in the cited guideline; ‘Cohort/implementation evidence’ indicates periodicity informed by published cohort or implementation data without a specific guideline recommendation; ‘Authors’ synthesis’ indicates periodicity proposed by the authors where neither guidelines nor cohort data specify an interval. This table does not itself constitute a graded clinical guideline; clinical judgment should adapt these periodicities to the individual case.

**Table 2:** Diagnostic technologies enabling combined viral hepatitis screening in STI settings.

Technology	Utility	Advantage	Limitation	Ideal Scenario	Maturity
Conventional serology (HBsAg, anti-HCV, 4th-gen HIV)	Backbone screening	Highly accurate, scalable, low cost	Requires confirmatory visit if not coupled to reflex	Universal first-line	Established
Reflex HCV-RNA	Same-sample confirmation of active HCV	Eliminates 2nd visit; speeds linkage and treatment	Requires lab algorithm and informatics integration	Any anti-HCV–positive sample	Established
Reflex anti-HDV/HDV-RNA	Detection of HBV–HDV coinfection in HBsAg-positives	Addresses major HDV under-diagnosis without new technology	Limited adoption; reagent and workflow setup needed	All HBsAg-positive samples	Established
Multiplex serological/molecular panels	Simultaneous HIV/HBV/HCV/syphilis testing	Reduced patient burden; aligns with shared exposure	Cost; regulatory and platform availability	STI clinics, outreach	Established
Point-of-care assays (incl. finger-stick HCV-RNA)	Same-day diagnosis at first contact	Decentralization; suitable outside hospital labs	Some loss of analytical sensitivity vs. central lab	Outreach, ED, prison, addiction services	Established
Dried blood spot (DBS)	Capillary sampling for HCV-RNA/HBV-DNA	No phlebotomy or cold chain; remote settings	Pre-analytical variability; lower sensitivity at low VL	Prisons, harm reduction, mobile clinics	Established
EHR alerts and best-practice notifications	Trigger screening at point of care	Reduces missed opportunities; scalable	Alert fatigue if poorly designed	Primary care, ED	Established
Self-sampling/self-testing	Patient-initiated screening	Bypasses stigma and access barriers	Linkage and quality assurance challenges	Hard-to-reach populations	Emerging
AI/machine learning risk stratification	Identify undiagnosed at-risk individuals from EHR data	Augments guideline-based screening	Algorithmic bias, data quality, equity concerns	Future/supportive role	Experimental

Abbreviations: ED, emergency department; EHR, electronic health record; STI, sexually transmitted infection; VL, viral load. Analytical performance parameters (e.g., limit of detection, cross-reactivity, and format-specific validation of dried blood spot versus plasma separation card samples) are not detailed in this table; see Section 4.3 for further discussion.

**Table 3:** Implementation barriers to integrated viral hepatitis diagnosis and proposed solutions.

Domain	Barrier	Proposed Solution	Recommendation Basis
Laboratory system	Reflex algorithms (especially anti-HDV/HDV-RNA) not configured; algorithms not audited	Standing institutional reflex protocols; periodic laboratory audit; double-reflex HDV pathway by default	**Evidence-based**—implementation tracking data [7,97]
Financing and reimbursement	Per-test billing favors fragmented testing; integrated panels not bundled	Bundled-panel reimbursement; cost-effectiveness modeling supports investment case	**Evidence-based**—cost-effectiveness studies [30,98]
Interoperability and EHR	Limited connectivity between STI clinics, hepatology, ID, primary care, microbiology	Shared EHR alerts and dashboards; linkage navigators with cross-service authority	**Authors’ synthesis**—no study tests this specific solution
Provider knowledge and workflow	Non-hepatology clinicians unfamiliar with HDV/reflex/elimination frameworks	Embedded best-practice alerts; brief structured training; clear responsibility for follow-up	**Mixed**—alerts evidence-based [26,99,100,101]; training component is authors’ synthesis
Stigma	Viral hepatitis associated with injection drug use, HIV, marginalized groups	Opt-out testing as default; STI- and PrEP-clinic-centered delivery; non-stigmatizing language	**Evidence-based**—opt-out testing literature [92,93,94,95,96,102,103,104,105,106]
Migration	Language, legal status, and access barriers limit specialist referral	Low-threshold community and migrant-health screening; cultural mediation; integrated linkage	**Authors’ synthesis**—refs [76,78] support the problem, not this specific solution
Prison settings	Short stays, fragmented continuity, variable infrastructure	Opt-out entry screening with reflex confirmation; in-prison treatment; coordinated post-release linkage	**Evidence-based**—prison implementation cohorts [107,108,109,110,111,112,113,114,115,116,117,118]
PrEP services	Variable integration of HBV/HCV/HDV testing alongside HIV and STIs	Standing reflex panels at every PrEP visit; periodicity per Table 1; HBV vaccination assessment	**Mixed**—PrEP cohort data supports periodicity; “standing panels” is authors’ synthesis
HDV-specific	Anti-HDV testing depends on individual clinician initiative	Mandatory institutional reflex anti-HDV in every HBsAg-positive sample; reflex HDV-RNA if positive	**Evidence-based**—reflex-implementation studies [97,119,120,121,122]
Equity and digital exclusion	AI/EHR-driven systems may exclude populations with poor digital footprint	Algorithm audit for bias; complementary outreach; preserve human navigation pathways	**Authors’ synthesis**—precautionary, general AI-bias literature [123,124,125,126,127]

Abbreviations: AI, artificial intelligence; EHR, electronic health record; HBsAg, hepatitis B surface antigen; HDV, hepatitis D virus; ID, infectious diseases; PrEP, pre-exposure prophylaxis; STI, sexually transmitted infection.

## Data Availability

All data discussed in this narrative review are derived from published literature and are publicly available through the cited sources. No new data were generated or analyzed in support of this review.
